# Shikonin-copper coordination nanoparticles for enhanced antibacterial and antibiofilm activity against *Staphylococcus aureus*

**DOI:** 10.1038/s41598-025-23269-4

**Published:** 2025-11-12

**Authors:** Yourang Jiang, Xueyong Tang, Ailin Wang, Hua Yang, Xue Jiang, Yinxin Zhang, Fanglu Lou

**Affiliations:** 1 Chongqing Clinical Research Center for Dermatology, Chongqing Key Laboratory of Integrative Dermatology Research, Key Laboratory of External Therapies of Traditional Chinese Medicine in Eczema, Department of Dermatology, Chongqing Traditional Chinese Medicine Hospital/The First Affiliated Hospital of Chongqing College of Traditional Chinese Medicine, Chongqing, 400011 China; 2 Chongqing No.6 People’s Hospital, Chongqing, 400060 China

**Keywords:** Shikonin, Antibacterial activity, Antibiofilm activity, Copper nanoparticles, Biological techniques, Biotechnology, Drug discovery

## Abstract

Antimicrobial resistance (AMR) poses a critical global health challenge, with an estimated 1.27 million AMR-attributable deaths in 2019 and projections of 39 million cumulative deaths from 2025 to 2050, particularly driven by *Staphylococcus aureus* and methicillin-resistant strains (MRSA) that form robust biofilms conferring up to 1000-fold antibiotic tolerance and complicating hospital-acquired infections. Here, we report a green, one-pot synthesis of shikonin-copper nanoparticles (SCu NPs), employing shikonin (SK)—a naphthoquinone from Lithospermum erythrorhizon roots-as a dual chelator and stabilizer, without exogenous reductants or surfactants; density functional theory (DFT) computations guide the design, predicting thermodynamically favored 1:2 Cu(II): SK stoichiometry, yielding stable spherical nanoparticles (39.25 ± 3.24 nm) with preserved Cu(II) oxidation state, as validated by TEM, XPS, XRD, and UV-Vis spectroscopy. SCu NPs exhibit potent antibacterial activity against *S. aureus* ATCC 25,923, with minimum inhibitory and bactericidal concentrations (MIC/MBC) of 4/8 µg/mL—half those of SK (8/16 µg/mL)—and rapid bactericidal kinetics, reducing viability by 66% within 2 h; antibiofilm assays reveal concentration-dependent inhibition, achieving up to 89% biomass reduction at 32 µg/mL, outperforming SK, CuSO_4_, and their mixture, with SEM confirming extensive membrane disruption and cytoplasmic leakage. This synergy arises from Cu(II)-mediated reactive oxygen species (ROS) generation, enhanced lipophilicity, and SK’s quorum-sensing inhibition, positioning SCu NPs as a sustainable, multi-target platform integrating natural product chemistry and nanotechnology to combat biofilm-associated AMR, mitigate resistance emergence, and advance therapeutics for recalcitrant infections.

## Introduction

Antimicrobial resistance (AMR) is a pressing global health crisis, with the World Health Organization and Global Research on Antimicrobial Resistance (GRAM) estimating 1.27 million AMR-related deaths in 2019 and projecting 39 million deaths from 2025 to 2050^1^. *S. aureus*, particularly methicillin-resistant strains (MRSA), drives hospital-acquired infections through robust biofilm formation, conferring up to 1,000-fold antibiotic resistance^2^ and complicating treatment in high-risk groups, such as ICU and immunocompromised patients^3^. This escalating threat underscores the urgent need for innovative antimicrobials that target both planktonic and biofilm-associated bacteria to mitigate AMR’s devastating impact^4^.

Copper, valued for centuries for its antimicrobial properties, exerts broad-spectrum bactericidal, virucidal, and antibiofilm effects via multi-target mechanisms, including reactive oxygen species (ROS) generation, membrane disruption, and enzymatic inhibition^5^. Clinical trials demonstrate copper-coated surfaces in healthcare settings reduce infection rates by up to 80%, highlighting its potential against resistant pathogens^6^. In recent years, copper nanoparticles (CuNPs) have emerged as highly versatile antimicrobial agents, gaining substantial research interest due to their unique physicochemical properties, including a high surface-to-volume ratio, tunable morphology and size, and controlled release of bioactive copper ions (Cu^2+^ and Cu^+^)^7,8^. These inherent characteristics empower CuNPs to breach bacterial cell envelopes with heightened efficiency, precipitating intracellular chaos through the induction of oxidative stress via reactive oxygen species (ROS) overproduction, irreversible protein misfolding and denaturation, and genomic DNA fragmentation, thereby culminating in bactericidal outcomes^9,10^. Biologically derived CuNPs and copper oxide nanoparticles (CuO NPs), in particular, have exhibited formidable antibacterial efficacy against multidrug-resistant Gram-positive pathogens, including methicillin-resistant Staphylococcus aureus (MRSA), by instigating membrane depolarization and permeabilization alongside ROS-mediated oxidative damage, frequently eclipsing the performance of legacy antibiotics in minimum inhibitory concentration (MIC) evaluations across diverse clinical isolates^11^. Systematic literature syntheses emphasize the expansive antimicrobial spectrum of CuO NPs, orchestrated via multifaceted mechanisms encompassing direct surface-mediated contact killing and sustained ionic leaching, which can achieve up to 99% eradication of viable S. aureus populations in controlled in vitro settings, underscoring their potential as adjunctive or standalone therapeutics^8^. Relative to silver nanoparticles (AgNPs), CuNPs confer notable advantages in terms of cost affordability and reduced environmental and mammalian cytotoxicity profiles, although they often demand synergistic integrations-such as bimetallic hybrids or polymer coatings-to rival AgNPs’ intrinsic antimicrobial vigor against entrenched resistant lineages^8^. Nonetheless, the therapeutic applicability of CuNPs against Gram-positive species like S. aureus remains curtailed by the resilient peptidoglycan matrix that impedes ion penetration, exacerbated by intrinsic vulnerabilities such as colloidal instability in aqueous milieus, facile particle aggregation leading to diminished surface reactivity, and expeditious oxidation of bioactive cuprous ions (Cu⁺) to thermodynamically stable yet less efficacious forms, collectively hampering their scalability and clinical deployment^8^. Mitigating these constraints necessitates the adoption of innovative stabilization modalities, including environmentally benign green synthesis routes leveraging plant-derived reductants and capping agents, as well as strategic complexation with organic ligands or biopolymers, to augment dispersion uniformity, orchestrate protracted ion release kinetics, potentiate antibiofilm interventions, and curtail off-target cytotoxicity toward eukaryotic cells^12^. However, copper’s efficacy against Gram-positive bacteria like *S. aureus* is hindered by thick peptidoglycan layers, and the instability of cuprous ions (Cu⁺)—prone to disproportionation and oxidation in aqueous environments-limits its clinical utility^13,14^. These challenges necessitate advanced formulations to enhance copper’s stability and bioavailability.

SK, a naphthoquinone extracted from Lithospermum erythrorhizon roots, exhibits potent antibacterial and antibiofilm activities by disrupting membrane integrity and suppressing quorum-sensing genes, such as the agr system in *S. aureus*^15^. Its ortho-quinone structure, featuring vicinal hydroxyl and carbonyl groups, provides ideal chelation sites for transition metal ions like copper, forming stable coordination complexes that amplify antimicrobial effects through synergistic ROS production and enhanced cellular penetration. Recent studies on metal-naphthoquinone complexes confirm their efficacy against Gram-positive biofilms by targeting matrix formation and intracellular pathways^16,17^. Given SK’s chelation potential, determining the optimal copper-SK stoichiometric ratio is critical to maximizing antimicrobial performance.

This study introduces a novel green synthesis strategy for SCu NPs, utilizing SK-a naturally derived naphthoquinone-as a dual chelating and stabilizing agent in a one-pot, pH-buffered reaction that eliminates the need for exogenous reductants or surfactants. Unlike conventional copper-based antimicrobial materials, which often exhibit aqueous instability, aggregation, and limited efficacy against robust Gram-positive biofilms like those of S. aureus, our approach innovatively integrates density functional theory (DFT) computations to predict and optimize 1:1 and 1:2 copper-SK coordination ratios. This rational, computation-guided design yields hybrid organic-inorganic nanostructures that maintain the Cu(II) oxidation state and enhance reactivity via synergistic reactive oxygen species (ROS) generation and membrane disruption. We employ DLS, TEM and in vitro antibacterial assays to assess SCu NPs’ antibacterial and antibiofilm performance against S. aureus. This work establishes a sustainable, multi-target antimicrobial platform that merges nanotechnology with natural product chemistry, advancing bioinspired nanomaterials to combat biofilm-associated infections and antimicrobial resistance while mitigating resistance emergence.

## Materials and methods

### Materials

All chemicals and reagents were purchased from Shanghai Aladdin Biochemical Technology Co., Ltd. (Shanghai, China), including Copper(II) sulfate pentahydrate (CuSO_4_·5H_2_O, Cat. No. C112396), Shikonin (SK, Cat. No. S119494), Vancomycin (Van, Cat. No.V301569), Dimethyl sulfoxide (DMSO, Cat. No. D103272), Nitric acid (69%, Cat. No. N112034), Acetonitrile (HPLC grade, Cat. No. A112035), Multi-element standards for ICP-OES (0.1–10 mg/L Cu in 2% HNO₃, Cat. No. M112355), Ethanol (absolute, Cat. No. E112004), Phosphate-buffered saline (PBS, powder, Cat. No. P274258), TSA (Tryptone Soy Agar, Cat. No. HB0177-2, Qingdao Hi-tech Industrial Park Hope Bio-technology Co., Ltd, China), Crystal violet (Cat. No. C112060), Glacial acetic acid (Cat. No. A112034), and Glutaraldehyde (25% in H_2_O, Cat. No. G105906). The bacterial strain Staphylococcus aureus ATCC 25,923 was obtained from Guangdong Microbial Culture Collection Center (China; originally sourced from ATCC, Cat. No. 25923).

### Synthesis of SCu NPs

SCu NPs were synthesized by the bottom-up method using CuSO_4_·5H_2_O as a metallic precursor. In a 25 mL flask, 5 mL of 5 mg/mL CuSO4·5H_2_O was prepared in 0.1 M Tris-HCl (pH 8.5). Then, 300µL of 10 mg/ml SK solution (DMSO) was added dropwise, followed by stirring for 4 h at room temperature. The resulting SK-copper coordination nanoparticles (SCu NPs) were purified by dialysis (MWCO: 12 kDa) against deionized water for 24 h, with water replacement every 6 h, to remove unbound Cu²⁺ ions, excess SK, DMSO residues, and buffer salts. The purified SCuNPs were stored in amber vials at 25℃, protected from light, until further use.

### SK and copper content quantification

SCu NPs were digested for elemental analysis and extracted for organic component quantification. For copper ion determination, 10 mg of lyophilized SCu NPs were suspended in 5 mL of 69% HNO₃,) and heated at 95 °C for 2 h in a microwave digestion system. The digest was cooled, diluted to 25 mL with ultrapure water, and filtered through a 0.22 μm PTFE membrane. For shikonin quantification, 10 mg of SCu NPs were dispersed in 2 mL of acetonitrile, sonicated for 30 min at 40 kHz, centrifuged at 12,000×g for 15 min, and the supernatant filtered for further analysis.

Copper content was quantified using inductively coupled plasma optical emission spectrometry (ICP-OES, PerkinElmer Avio 500)^12^. Instrument parameters included RF power of 1.3 kW, plasma gas flow of 12 L/min argon, auxiliary gas flow of 0.5 L/min, nebulizer gas flow of 0.7 L/min, and sample uptake rate of 1 mL/min. The emission line for copper was monitored at 324.752 nm. Calibration was performed with multi-element standards (0.1–10 mg/L Cu in 2% HNO₃). Samples were analyzed in triplicate, with limits of detection (LOD) and quantification (LOQ) of 0.5 µg/L and 1.5 µg/L, respectively. Copper loading efficiency was calculated as (measured Cu mass / theoretical Cu mass)×100%.

Concentration of SK was determined by high-performance liquid chromatography (HPLC; Agilent 1260 Infinity II) with UV detection^12^. A reversed-phase C18 column (Thermo Hypersil GOLD, 250 × 4.6 mm, 5 μm) was used at 25 °C. The mobile phase comprised acetonitrile:0.1% acetic acid (47:53, v/v), delivered at 1.0 mL/min. Detection was at 280 nm, with an injection volume of 20 µL. Calibration curves were constructed using shikonin standards (0.1–100 µg/mL). Samples were analyzed in triplicate, with LOD and LOQ of 0.05 µg/mL and 0.15 µg/mL, respectively. Shikonin encapsulation efficiency was calculated as (mass of measured SK / mass of initial SK )×100%.

### Characterization of SCu NPs

The hydrodynamic diameter, polydispersity index (PDI), and zeta potential of SCu NPs were determined by dynamic light scattering (DLS, NanoBrook Omni, Brookhaven Instruments, USA). To assess colloidal stability, these parameters were monitored over time in 10% fetal bovine serum (FBS) at days 0, 7, 14, and 21 at room temperature. Morphological features of SCu NPs were examined using scanning electron microscopy (SEM, SU8010, Hitachi, Japan) and transmission electron microscopy (TEM, Hitachi HT7700, Japan). Prior to SEM/TEM imaging, the nanoparticles were lyophilized at − 80 °C for 48 h and subsequently redispersed in ethanol via ultrasonication. Elemental mapping and composition analysis were performed via energy-dispersive X-ray spectroscopy (EDX, attached to SEM, Oxford Instruments, UK). Fourier-transform infrared (FTIR) spectra of SK and freeze-dried SCu NPs were recorded on a Nicolet iS50 spectrometer (Thermo Fisher Scientific, USA) across 4000–400 cm^− 1^. UV-vis absorption profiles (230–800 nm) were acquired using a UV-2600 spectrophotometer (Shimadzu, Japan). Thermogravimetric analysis (TGA) was conducted under nitrogen flow (30–800 °C, 10 °C·min⁻¹ heating rate) via a TGA5500 instrument (TA Instruments, USA). X-ray diffraction (XRD) patterns were collected on a D8 Advance diffractometer (Bruker, Germany) with Cu Kα radiation (λ = 1.5406 Å, 5–80° 2θ range).

### DFT calculations

To further determine the optimal stoichiometric ratio of the complexes, we conducted computational simulations using Gaussian 09 software with Density Functional Theory (DFT). Geometry optimizations and frequency calculations were performed at the B3LYP/6-311G(d, p) level^18^, while single-point energy calculations were carried out using the B3LYP/6-311 + G(d, p) basis set. The SMD continuum solvation model was employed with water as solvent^19^. To improve computational accuracy, Grimme’s GD3 empirical dispersion correction^20^ was implemented, and thermodynamic free energies were obtained through Shermo software^21^. We systematically investigated SK-Cu complexes with 1:1 and 2:1 molar ratios. For in-depth analysis of non-covalent interactions in the optimal configurations, we performed Independent Gradient Model based on Hirshfeld partition (IGMH) analysis using the IRI method^22^ implemented in Multiwfn software^23^, with visualization conducted through VMD^24^. Additionally, frontier molecular orbital (HOMO-LUMO) analyses were performed to evaluate the chemical reactivity of these complexes.

### Antibacterial efficiency

#### Determination of minimum inhibitory concentration (MIC) and minimum bactericidal concentration (MBC)

S. aureus was cultured on TSA at 37 °C for 24 h. Single colonies were suspended in phosphate-buffered saline (PBS) to achieve a turbidity equivalent to a 0.5 McFarland standard (approximately 1.5 × 10^8^ CFU/mL) and further diluted 1:200 in PBS to yield a final inoculum density of approximately 5 × 10^5^ CFU/mL. SK was dissolved in dimethyl sulfoxide (DMSO) to a stock concentration of 1024 µg/mL. Both SCu NPs and CuSO₄·5H₂O were suspended in PBS to stock concentrations of 1024 µg/mL (with SCu NPs adjusted based on total mass, and CuSO₄·5H₂O based on copper equivalence, considering the predicted molar ratio of SK: Cu at 2:1). For the SK་CuSO₄·5H₂O mixture, individual stock solutions of SK (1024 µg/mL in DMSO) and CuSO₄·5H₂O (1024 µg/mL in PBS, copper-equivalent) were combined in a 2:1 molar ratio (SK: Cu) to achieve equivalent concentrations to those in SCu NPs, and the mixture was freshly prepared and vortexed for homogeneity prior to dilution. Vancomycin (Sigma-Aldrich, USA) was used as a positive control and dissolved in sterile water to a stock concentration of 1024 µg/mL. Two-fold serial dilutions of SK, SCu NPs, CuSO₄·5H₂O, SK་CuSO₄·5H₂O mixture, or vancomycin (ranging from 0.5 to 1024 µg/mL, with adjustments for copper equivalence in SCu NPs, CuSO₄·5H₂O, and the mixture) were prepared in 96-well microtiter plates (Corning, USA), with each well containing 100 µL of the antimicrobial solution and 100 µL of bacterial inoculum. Plates were incubated statically at 35 °C for 24 h. The MIC was defined as the lowest concentration preventing visible bacterial growth, confirmed by an optical density at 600 nm using a microplate reader (Synergy H1, BioTek, USA). For MBC determination, 10 µL aliquots from wells at and above the MIC were spotted onto TSA plates and incubated at 35 °C for 24 h. The MBC was defined as the lowest concentration resulting in ≥ 99.9% reduction in viable CFU relative to the initial inoculum. All experiments were performed in triplicate. For synergy assessment, a checkerboard assay was employed for the SK་CuSO₄·5H₂O mixture: serial dilutions of SK (along rows) and CuSO₄·5H₂O (along columns) were combined in a 96-well plate at ratios equivalent to the 2:1 molar ratio, with the fractional inhibitory concentration index (FICI) calculated as FICI = (MIC_SK in combo / MIC_SK alone)་(MIC_Cu in combo / MIC_Cu alone), where FICI ≤ 0.5 indicates synergy.

#### Time-kill kinetics assay

For time-kill kinetics, *S. aureus* was cultured in TSB at 37 °C with shaking (200 rpm) for 5 h to reach exponential phase, then adjusted to 10^6^ CFU/mL. SK (dissolved in DMSO), SCu nanoparticles (suspended in PBS), CuSO_4_·5 H₂O (suspended in PBS, at copper-equivalent concentrations), and the SK + CuSO₄·5 H₂O mixture (prepared by combining individual stock solutions in a 2:1 molar ratio [SK: Cu] to match equivalent concentrations in SCu NPs, freshly vortexed for homogeneity) and vancomycin (dissolved in sterile water, as a positive control) were evaluated. Concentrations were set at 16 µg/mL for SK, SCu NPs, and the mixture (total mass equivalence; corresponding to 2×MIC for SK and 4×MIC for SCu NPs), 512 µg/mL for CuSO₄·5 H₂O (copper equivalence, escalated due to lack of effect at lower doses), and 4 µg/mL for vancomycin (4×MIC) to assess rapid bactericidal kinetics beyond minimal inhibition, in accordance with CLSI guidelines. Bacterial suspensions were incubated with each antimicrobial agent at 37 °C with gentle shaking. At designated time points (2, 4, and 6 h), 100 µL aliquots were serially diluted (10^4^- and 10^6^-fold) in PBS, spread-plated (100 µL) onto TSA, and incubated at 37 °C for 24 h to quantify viable CFU/mL. Untreated controls were processed in parallel, and results were expressed as survival percentages relative to these controls. For scanning electron microscopy (SEM), treated and control bacterial suspensions (after 6 h incubation) were centrifuged, washed three times with PBS, fixed in 2.5% glutaraldehyde at 4°C overnight, dehydrated through an ascending ethanol series (30%, 50%, 70%, 90%, and 100%; 15 min each), sputter-coated with gold, and imaged using a TESCAN VEGA3 SEM (Czech Republic) at 10 kV accelerating voltage.

### Anti-biofilm effects

Biofilm formation inhibition assays were performed using the crystal violet (CV) staining method in flat-bottom 96-well polystyrene microtiter plates (Corning, USA). *Staphylococcus aureus* was cultured overnight in tryptic soy broth (TSB) supplemented with 1% (w/v) glucose at 37 °C with shaking (200 rpm), adjusted to a turbidity equivalent to a 0.5 McFarland standard (approximately 1.5 × 10⁸ CFU/mL), and diluted 1:100 in fresh PBS to yield an inoculum of approximately 10^6^ CFU/mL. Aliquots (100 µL) of the bacterial suspension were dispensed into wells and co-cultured with 100 µL of the test agents-SK (dissolved in DMSO, final concentration < 1% v/v), CuSO_4_·5H_2_O (suspended in PBS, copper-equivalent), SK་CuSO_4_·5 H₂O mixture (freshly prepared at a 2:1 molar ratio [SK: Cu] for SCu nanoparticle [NP] equivalence, vortexed for homogeneity), or SCu NPs (suspended in PBS, total mass equivalence)—at two-fold serial dilutions from 0.5 to 64 µg/mL (adjusted for equivalence as detailed in Sect. 2.4 for MIC/MBC assays). Vancomycin (dissolved in sterile water) served as the positive control at equivalent concentrations. Plates were incubated statically at 37 °C for 24 h. Untreated wells with bacterial inoculum and the corresponding vehicle (DMSO for SK treatments, PBS for CuSO₄·5H_2_O and SCu NP treatments, a DMSO-PBS mixture for combination treatments, or sterile water for vancomycin treatments) at equivalent concentrations served as negative controls (100% biofilm formation). Following incubation, non-adherent cells were removed by three washes with 0.01 M PBS (pH 7.4). Biofilms were fixed with 99% methanol for 15 min, air-dried, stained with 0.1% (w/v) CV for 20 min, rinsed with distilled water, and solubilized in 33% (v/v) glacial acetic acid. Biofilm biomass was quantified by OD at 590 nm (OD_590_) using a microplate reader (Synergy H1, BioTek, USA). Inhibition (%) was calculated as [1 - (OD_590 treated_ / OD_590 negative control_)]×100. The minimum biofilm inhibitory concentration (MBIC) was defined as the lowest concentration yielding ≥ 80% biomass reduction relative to negative controls. Assays were conducted in triplicate, with data presented as means ± SD.

### Statistical analysis

Statistical analyses were conducted using SPSS 24.0 software (IBM). Statistical differences (*p < 0.05) were determined using one-way ANOVA followed by Bonferroni post hoc test for multiple comparisons. In all cases, differences were considered significant if *p* < 0.05. Results were expressed as mean ± standard deviation (SD) from five independent biological replicates.

## Results and discussion

### The synthesis and characterization of SCu NPs

**Fig. 1:**
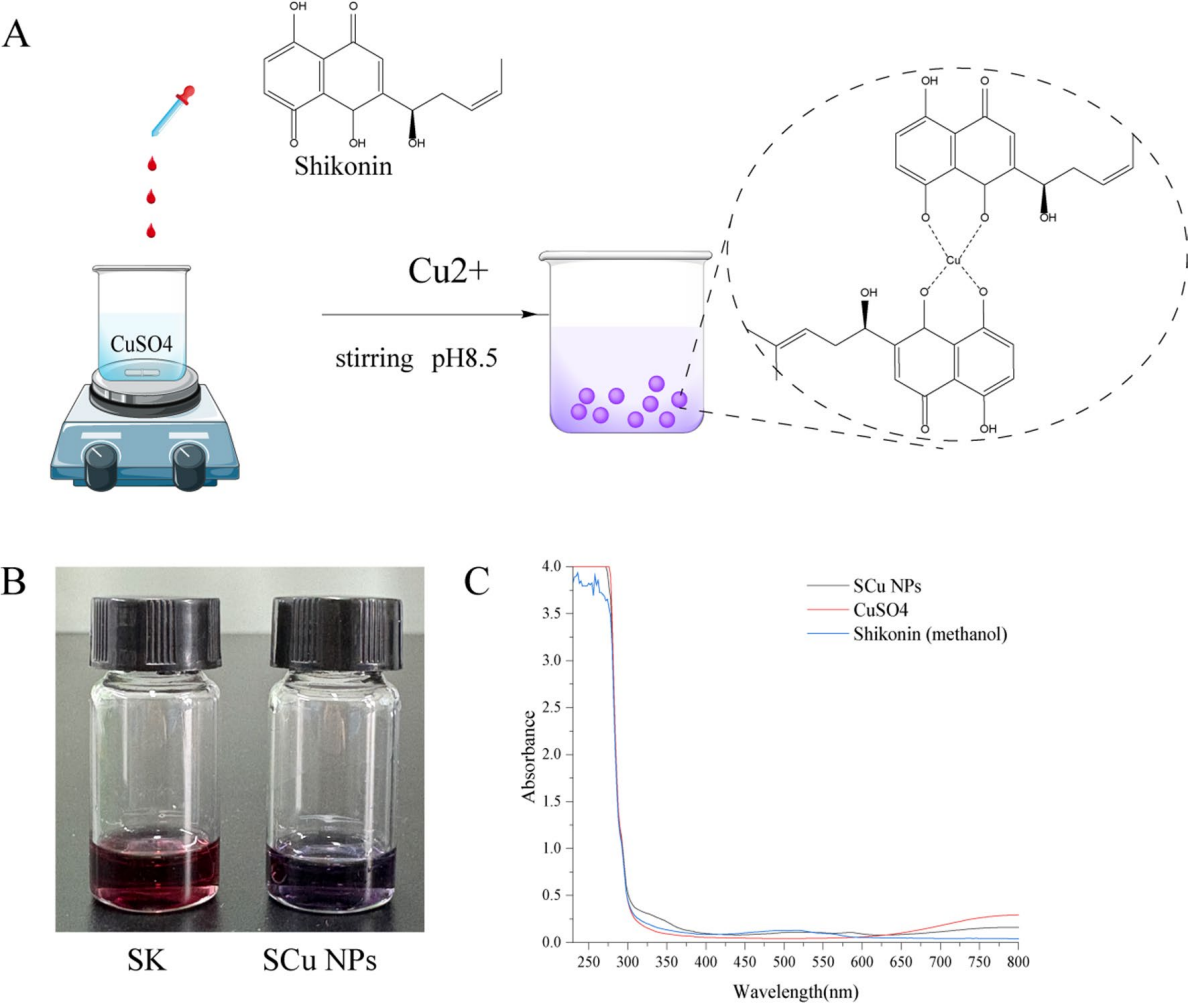
Schematic of the preparation, morphology and UV-Vis spectrum of the SCu NPs. (**A**) Formation mechanism of SCu NPs from SK under alkaline conditions; (**B**) Overall morphology of SK in methanol and SCu NPs in water; (**C**) UV-Vis spectrum of CuSO_4_, SK and SCu NPs.

SK, a naphthoquinone derivative bearing hydroxyl and quinone functionalities, exhibits robust antioxidant and reductive properties, with pH-dependent enhancement under alkaline conditions. Its antioxidant capacity arises from proton and electron donation to neutralize free radicals, positioning SK as an effective ligand for metal ion coordination^25^. SK also engages in redox cycling via interaction with thioredoxin reductase 1 (TrxR1), generating superoxide anions and demonstrating its electron transfer capability^26^. In alkaline environments (pH 8.5), SK’s hydroxyl and quinone groups likely coordinate Cu2 + ions through chelation, forming stable copper-SK complexes and the main mechanism can be illustrated in Fig. 1A. The quinone ring may facilitate single-electron transfer within the redox cycle, while the resultant oxidized SK stabilizes the complex via coordination of hydroxyl or carbonyl groups with Cu2+, preventing aggregation. This chelation mechanism aligns with studies on naphthoquinone-copper(II) complexes^27^, where oxygen atoms from hydroxyl and quinone groups form stable coordination bonds with Cu²⁺, similar to the octahedral or square-planar geometries observed in related compounds such as lawsone-Cu(II) complexes^28^. However, unlike previous reports on reductive synthesis of Cu(0) nanoparticles using plant extracts^12^, our approach leverages SK’s pH-dependent reductive properties without complete reduction to metallic Cu, resulting in coordination nanoparticles rather than zero-valent metal particles. This distinction highlights SK’s dual role as both a reductant and stabilizer, advancing beyond traditional polyphenol-based syntheses^12^ by incorporating quinone-mediated electron transfer.

At pH 8.5, SK reacted with CuSO4 to form shikonin-copper coordination nanoparticles (SCu NPs), as evidenced by a striking color shift from wine red to deep purple (Fig. 1.B) and enhanced UV-vis absorption in the ultraviolet region (Fig. 1.C), signaling Cu(II)-quinone coordination rather than reductive formation of Cu(0) nanoparticles^29^.

Notably, the UV-vis spectrum of SCu NPs lacks the characteristic peak typically observed around 550–600 nm for zero-valent Cu nanoparticles^30^, which is an unexpected finding compared to conventional reductive syntheses. This absence is attributed to the formation of Cu(II)-quinone coordination complexes rather than metallic Cu(0) particles, as evidenced by the enhanced absorption in the ultraviolet region (broadened peaks at ~ 280 nm and ~ 350 nm, indicative of ligand-to-metal charge transfer (LMCT) bands)^30^. Such spectral features are consistent with reported Cu(II)-polyphenol complexes^31^, where chelation stabilizes the + 2 oxidation state and prevents aggregation or further reduction. This mechanism not only explains the color shift from red to deep purple (Fig. 1B) but also underscores SK’s efficacy in modulating metal ion valence under alkaline conditions, differing from acidic environments where reduction to Cu(0) might predominate^31^.

**Fig. 2:**
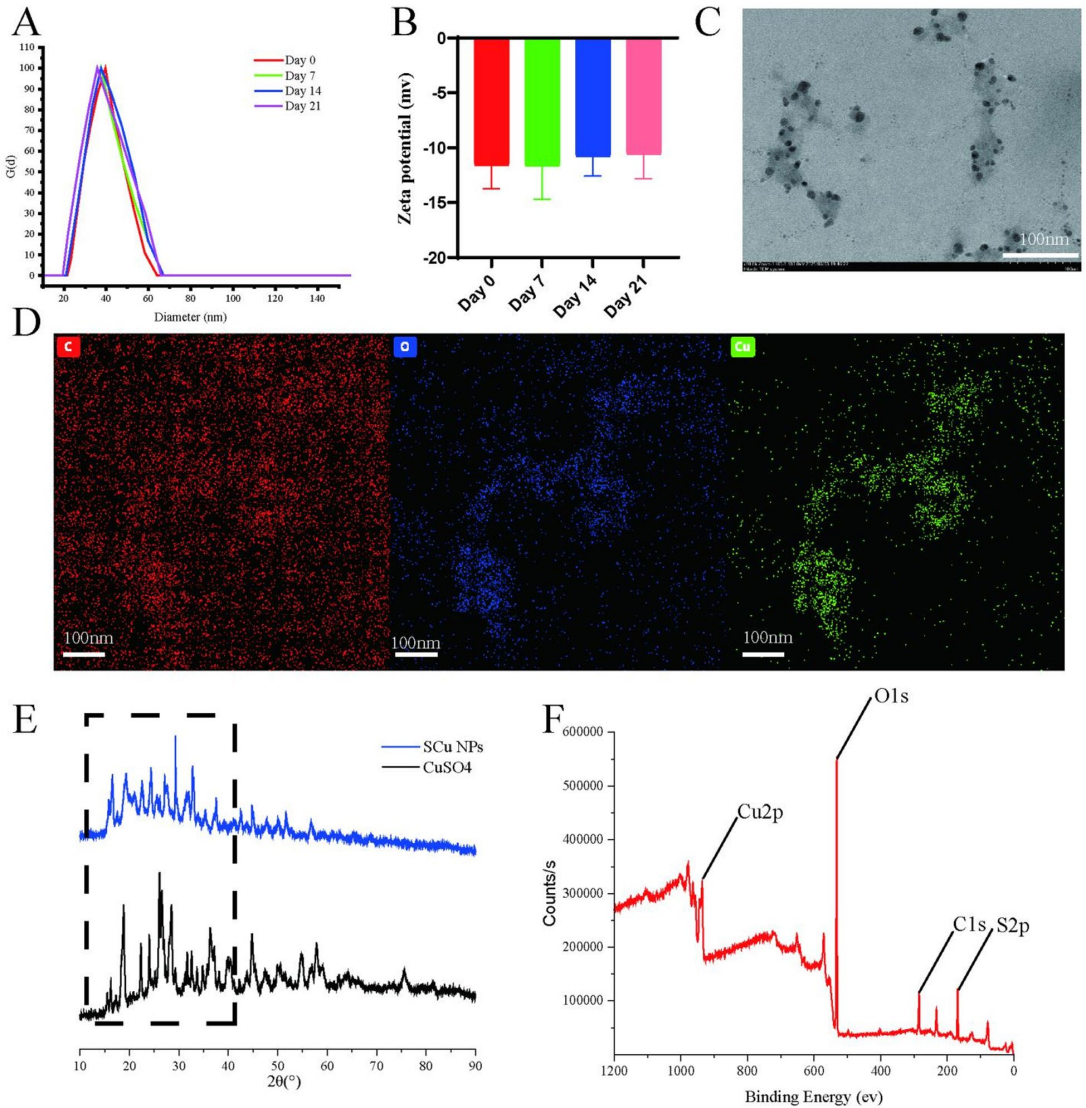
Characterization of SCu NPs. (**A**,**B**) Hydrodynamic size distribution and of zeta potential SCu NPs at day 0, day 7, day 14, and day 21, determined by dynamic light scattering (DLS) (*n* = 3). (**C**) TEM images showing the morphology of SCu NPs. (**D**) Energy-dispersive X-ray spectroscopy (EDS) elemental mapping of SCu NPs. (**E**) XRD pattern of SCu NPs. (**F**) XPS survey spectrum of SCu NPs.

The synthesis of SCu NPs via the bottom-up coordination approach yielded stable nanoparticles with a measured copper loading efficiency of 4.8 ± 0.9% (*n* = 3), as determined by ICP-OES following microwave-assisted nitric acid digestion. This low efficiency aligns with the excess CuSO4 precursor employed (approximately 10-fold molar excess relative to SK) and the thorough elimination of unbound ions through dialysis. HPLC analysis after acetonitrile extraction demonstrated a high encapsulation efficiency of SK at 95.5 ± 2.3% (*n* = 3), suggesting near-quantitative integration of the limiting ligand into the nanoparticle structure, presumably facilitated by robust bidentate chelation through its dihydroxy naphthoquinone groups. Based on the quantified contents, the molar ratio of SK to Cu in the purified SCu NPs was determined to be 1.92 ± 0.14 : 1. UV–Vis spectral data do not show the characteristic surface plasmon resonance peak of copper nanoparticles (~ 593 nm)^32^, suggesting that metallic Cu⁰ nanoparticles were not formed. The absorption pattern supports the formation of coordination complexes^33^, not classical metallic nanoparticles. Dynamic light scattering (DLS) revealed a hydrodynamic diameter of 39.25 ± 3.24 nm (PDI 0.22) (Fig. 2.A), indicating uniform sizing, and a zeta potential of -11.63 ± 2.11 mV (Fig. 2.B), reflecting moderate colloidal stability that could be enhanced through surface modification, unlike highly stable CuO nanoparticles (> 30 mV)^34^.To assess long-term structural integrity, we monitored these parameters in 10% FBS over time at room temperature. Remarkably, no significant differences were observed (*p* > 0.05) in hydrodynamic diameter or zeta potential at day 7 (36.42 ± 4.62 nm, -11.64 ± 3.05 mV), day 14 (39.84 ± 2.47 nm, -10.79 ± 1.81 mV), and day 21 (38.92 ± 7.78 nm, -10.57 ± 2.27 mV)(Fig. 2A and B).This temporal consistency highlights the robust stability of the SCu NPs, likely stemming from the protective Cu(II)-quinone coordination complexes that mitigate aggregation and oxidation, thereby supporting their potential for sustained applications in antimicrobial contexts without rapid degradation. TEM confirmed spherical SCu NPs (around 20 nm) (Fig. 2C) smaller than DLS results due to hydration layers, underscoring the complementary nature of these techniques^35^. Energy-dispersive X-ray spectroscopy (Fig. 2D) establishes coexistence of carbon, copper, and oxygen species. XRD showed SCu NPs with intensified peaks at 15.4°, 18.6°, and 24.0° (124,883–150,733 counts) versus CuSO4(15.3°, 18.3°, 23.9°; 24,750 − 49,716 counts), and broad peaks at 16.2°–18.8° (Fig. 2.E), indicating nanoscale crystallites (10–20 nm) in an amorphous SK matrix, distinct from purely inorganic metallic copper nanoparticles^30^.

XPS detected Cu(II) (Cu 2p at 935 eV, satellites at 940–945 eV), residual sulfate (S 2p at 168–169 eV), and SK’s organic framework (C 1s at 284 eV, O 1s at 532 eV) (Fig. 2F), confirming a stable coordination complex without reduction, with sulfate possibly integrated as a co-ligand27,28. Importantly, the absence of a surface plasmon resonance peak at ~ 593 nm in the UV-Vis spectrum (Fig. 1C) further corroborates that SCu NPs are not classical metallic Cu⁰ nanoparticles but rather Cu(II)-SK coordination nanoparticles, consistent with the chelation mechanism involving SK’s quinone moiety^36,37^. The alkaline pH likely deprotonated SK’s quinone hydroxyls, enhancing Cu(II) chelation and preventing reduction, contrasting with polyphenol-mediated reductions to Cu(0)^38^. This hybrid organic-inorganic architecture, integrating SK’s stabilizing matrix with Cu(II)’s redox-active sites, positions SCu NPs for catalytic applications, leveraging enhanced selectivity from organic ligands^39^. The nuanced coordination chemistry, preserving Cu(II) amidst SK’s redox-active quinone, highlights a sophisticated interplay of ligand-metal interactions; however, the precise stoichiometry and coordination geometry remain underexplored due to limited studies on Cu(II)-SK complexes.

### DFT calculations

**Fig. 3:**
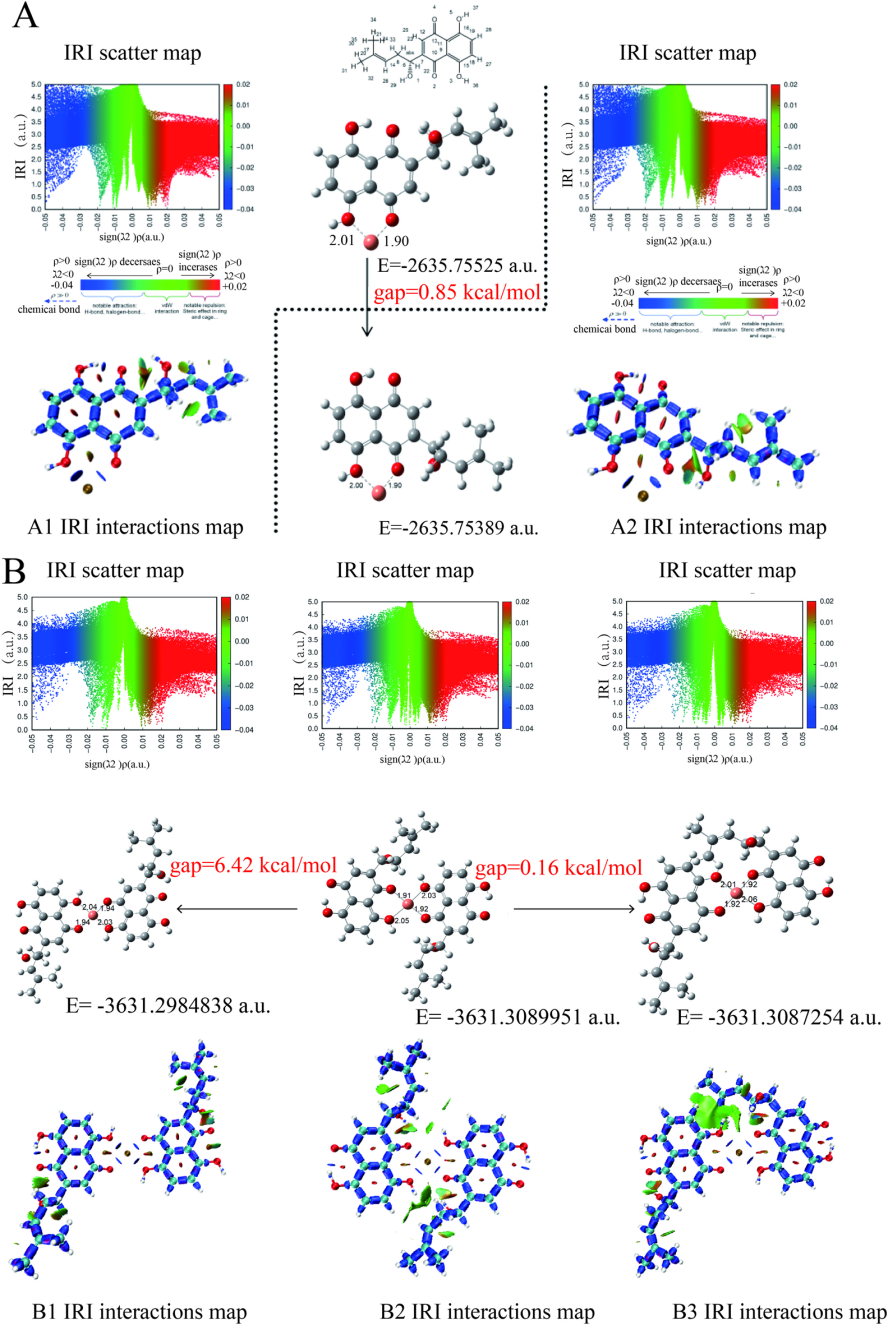
Analysis of copper-SK interaction sites and intramolecular interactions at 1:1 (**A**) and 1:2 (**B**) Stoichiometries. The IRI (interacting region indicator) scatter maps for SCu NPs at 1:1 (**A**) and 1:2 (**B**) ratios spatially resolve intramolecular interactions, with color coding defined as follows: blue regions correspond to covalent bonds, ionic bonds, and hydrogen bonds; green regions represent van der Waals forces; red regions indicate steric repulsions.

**Fig. 4:**
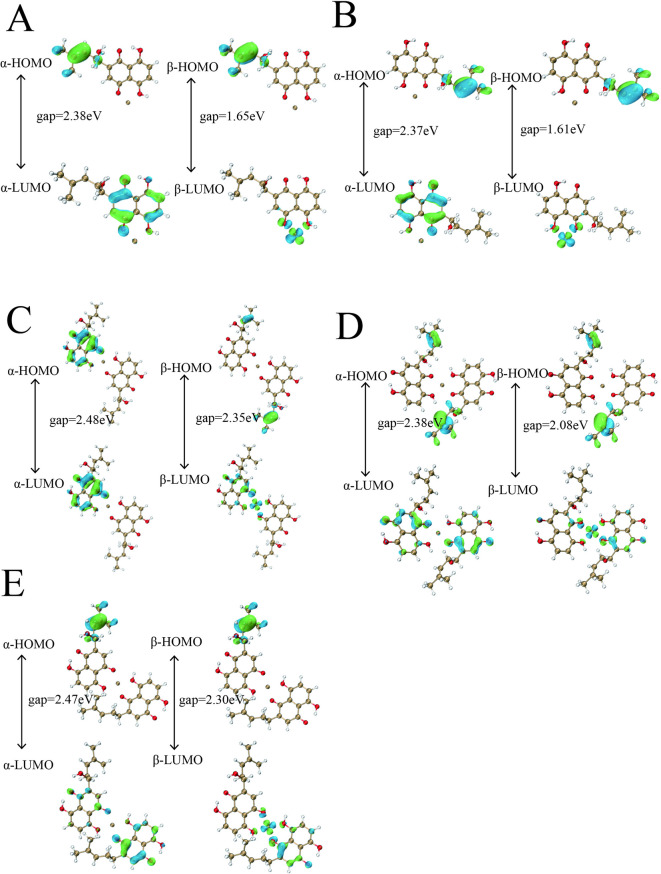
Comparative HOMO-LUMO analysis of four Cu2+-SK coordination configurations. The corresponding configurations of the SK-copper complexes for positions (**A**–**E**) are designated as A1, A2, B1, B2, and B3 respectively.

Density functional theory (DFT) calculations elucidate the coordination of Cu²⁺ with SK in aqueous solution (Fig. 3), revealing two 1:1 binding modes via the C4 carbonyl/C5 hydroxyl (A1) and C2 carbonyl/C3 hydroxyl (A2) regions (Fig. 3.A), with a free energy difference of only 0.85 kcal/mol, indicating dynamic equilibrium at ambient conditions^40^. Interacting region indicator (IRI) analysis confirms dominant ionic interactions, consistent with the strong chelating ability of shikonin’s quinone moiety. This near-equivalent stability aligns with copper(II)-naphthoquinone complexes, such as Cu(II)-lawsone, where oxygen atoms form stable bidentate chelates^29^. In 1:2 coordination, three geometries (B1–B3) emerge, with the B2 configuration exhibiting the lowest free energy (ΔG = -6.42 kcal/mol relative to B1) (Fig. 3.B), driven by symmetric binding of two SK molecules at C2/C3 sites, stabilized by van der Waals interactions and weak hydrogen-bond networks^39^. The marginal 0.16 kcal/mol difference between B2 and B3 suggests their coexistence, enhancing structural diversity in SCu NPs. HOMO-LUMO analysis (Fig. 4) reveals B2’s smallest band gap (Fig. 4.D) compared to that of others (Fig. 4.A-C and E), implying the highest chemical softness (inverse of bandgap per Koopmans’ theorem) and potentially superior reactivity despite its thermodynamic stability. This is corroborated by studies on Cu(II)-quinone complexes, where reduced bandgaps may enhance charge transfer for catalytic applications^41^. The predominance of the 1:2 B2 configuration may contribute to the amorphous SK matrix embedding nanoscale crystallites observed in XRD, with XPS confirming Cu(II) retention due to robust quinone coordination, distinguishing SCu NPs from reduced Cu(0) systems. While speculative, the synergistic stability and reactivity of B2 suggest a potential role in positioning SCu NPs as promising candidates for antimicrobial applications, leveraging Cu(II)’s redox activity within SK’s organic framework, akin to Cu(II)-Schiff base complexes^30^.Further experimental studies are warranted to directly link these electronic properties to biological efficacy.

### Antibacterial activity of SCu NPs

The SCu NPs exhibited superior antibacterial activity against *S. aureus* compared to SK alone, as evidenced by MIC and MBC values (4 µg/mL and 8 µg/mL versus 8 µg/mL and 16 µg/mL, respectively). This augmentation is likely attributable to the coordinated SK-Cu structure, which facilitates enhanced membrane disruption and reactive oxygen species (ROS) generation. Although the SK་CuSO₄·5 H₂O mixture demonstrated no improvement over SK alone (MIC/MBC: 8/16 µg/mL), checkerboard assays revealed synergistic interactions, with a fractional inhibitory concentration index (FICI) of 0.375, suggesting that Cu²⁺ ions potentiate SK activity in the free form—albeit less effectively than in the nanoparticulate coordinated configuration. CuSO_4_·5H_2_O alone displayed weak antibacterial potency (MIC/MBC: 128/256 µg/mL), while vancomycin, employed as the positive control, confirmed standard efficacy (MIC/MBC: 1/2 µg/mL), thereby validating the experimental setup.

**Fig. 5:**
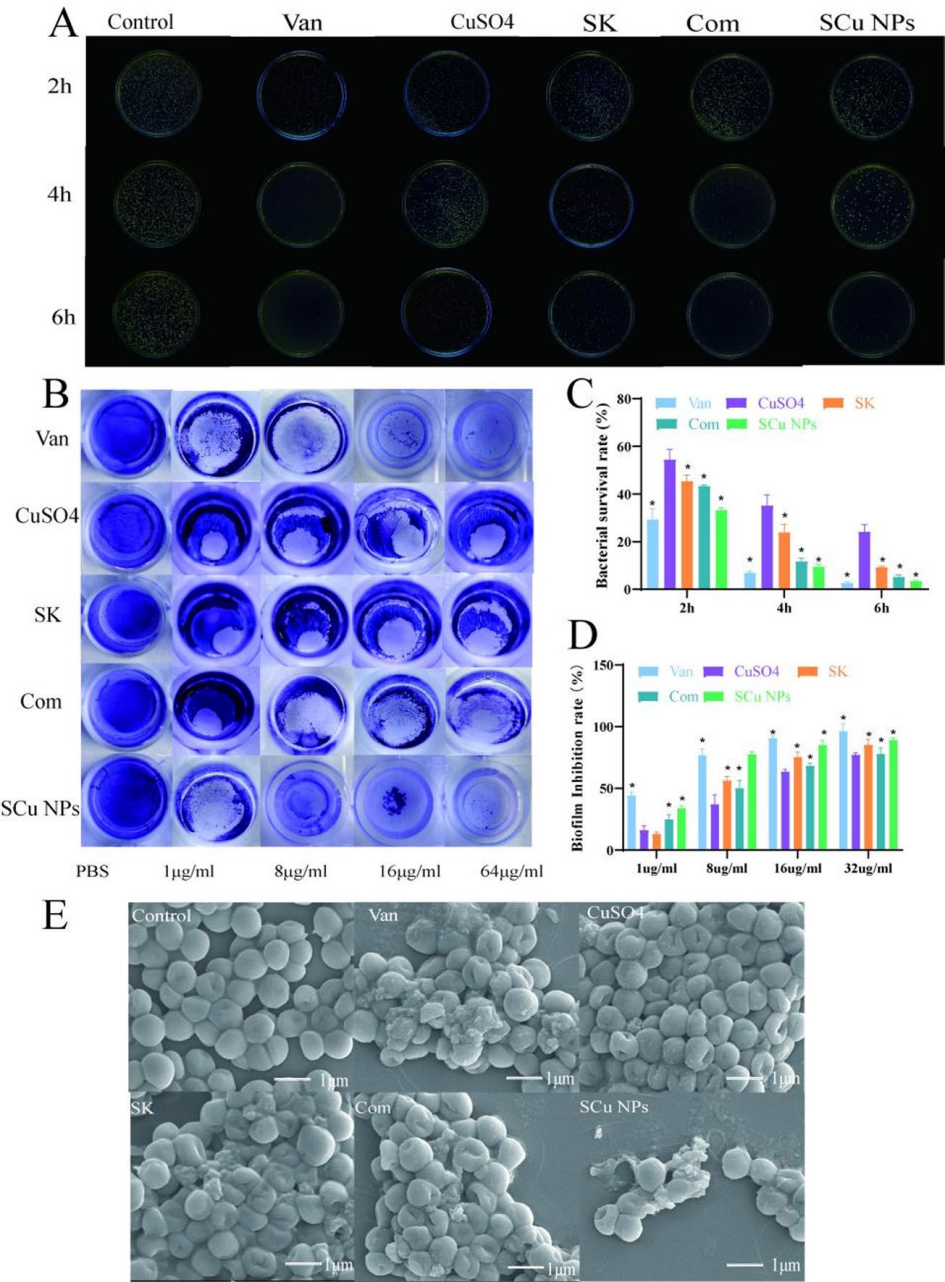
Antibacterial and antibiofilm performance of SK and SCu NPs against *S. aureus*. (**A**) Bacterial colonies on TSA plates against *S. aureus* after incubation for 2, 4, and 6 h at 37 °C. (**B**) Antibiofilm inhibition rate against *S. aureus* after incubation for 48 h at 37 °C. (**C**) Bacterial survival rate against *S. aureus* after incubation for 2, 4, and 6 h at 37 °C. (*n* = 3,*p < 0.05 vs. CuSO4-treated control group). (**D**) Antibiofilm effects against *S. aureus* after incubation for 48 h at 37 °C (*n* = 3,*p < 0.05 vs. CuSO4-treated control group). (**E**) Scanning electron microscopy (SEM) images depicting cell morphology of *S. aureus* after 6 h incubation at 37 °C.

The antibacterial and antibiofilm efficacy of SCu NPs against *S. aureus* was evaluated in comparison with Van, CuSO4, SK, and Com. Plate counting assays revealed time-dependent reductions in bacterial viability across all treatments (Fig. 5A, C). At 2 h, SCu NPs achieved the lowest survival rate of 33.58 ± 1.05%, outperforming SK (45.46 ± 1.96%, *p* < 0.05) and Com (43.35 ± 0.43%, *p* < 0.05), while Van and CuSO4 yielded 29.38 ± 3.69% and 54.50 ± 3.40%, respectively. By 4 h, survival rates further declined, with SCu NPs at 9.52 ± 0.89%, comparable to Van (7.32 ± 0.48%) and superior to SK (23.87 ± 2.86%, *p* < 0.05). At 6 h, SCu NPs reduced survival to 3.42 ± 0.27%, nearing Van’s 2.58 ± 0.45% and markedly lower than SK (9.31 ± 0.29%, *p* < 0.05) and CuSO4 (24.15 ± 2.22%, *p* < 0.05). These kinetics indicate that SCu NPs exhibit rapid bactericidal activity, likely enhanced by chelation, surpassing the additive effects observed with Com.

Biofilm inhibition was assessed via crystal violet staining after 48 h incubation (Fig. 5B, D). SCu NPs demonstrated concentration-dependent efficacy, achieving 33.86 ± 2.22% inhibition at 1 µg/mL, escalating to 89.17 ± 1.89% at 32 µg/mL. This outperformed SK across all concentrations (13.15 ± 1.48% at 1 µg/mL, 56.34 ± 3.23% at 8 µg/mL, 75.47 ± 3.78% at 16 µg/mL, and 85.17 ± 3.69% at 32 µg/mL, *p* < 0.05 at 1–16 µg/mL), and was competitive with Van (44.22 ± 2.79% at 1 µg/mL to 96.39 ± 5.86% at 32 µg/mL). Notably, Com showed intermediate activity (27.55 ± 1.82% at 1 µg/mL to 84.63 ± 4.12% at 32 µg/mL), suggesting synergy in the nanoparticle form, while CuSO4 exhibited the weakest inhibition (16.16 ± 3.49% at 1 µg/mL to 77.22 ± 1.80% at 32 µg/mL, *p* < 0.05 vs. SCu NPs at all concentrations). SEM corroborated these findings (Fig. 5E), which the control *S. aureus* appeared spherical and intact with smooth surfaces. Van treatment caused bacterial rupture with extrusion of intracellular contents. SK and Com treatments induced similar morphological changes, including membrane disruption and cytoplasmic leakage. SCu NPs treatment resulted in the most severe damage, with extensive cytoplasmic leakage, pronounced membrane collapse, and deformed, shrunken cellular morphology.

This rapid efficacy likely arises from copper-SK chelation, where copper’s redox activity (Cu²⁺/Cu⁺, 0.16 V) generates ROS to cause oxidative damage, while SK disrupts membrane integrity and biofilm gene expression^15,42^. Recent studies corroborate this, showing that copper-based chelates disrupting *S. aureus* biofilms through ROS and quorum-sensing inhibition^16,43^. In contrast, copper-sorbate chelates achieved only 8.7% biofilm adhesion inhibition in *S. aureus* under different conditions^44^, whereas SCu NPs’ superior 2-hour efficacy likely stems from SK’s naphthoquinone structure enhancing copper’s bioactivity through increased lipophilicity.

## Discussion

The green synthesis of SCu NPs via a one-pot, pH-buffered (8.5) reaction exemplifies a paradigm in sustainable nanomaterial fabrication, exploiting shikonin’s (SK) naphthoquinone scaffold as a dual chelating ligand and stabilizer to yield stable Cu(II)-based nanostructures without exogenous reductants or surfactants, thereby addressing the aggregation and oxidation vulnerabilities of conventional CuNPs in aqueous milieus^31^. Characterization reveals a hydrodynamic diameter of 39.25 nm (PDI 0.22) and zeta potential of -11.63 mV by DLS, with TEM confirming spherical morphology (10–20 nm) and stability over 21 days, contrasting with biosynthesized CuO NPs from plant extracts that often exhibit PDI > 0.3 and aggregation within days due to inadequate capping^12^.The UV-Vis absence of a ~ 593 nm plasmon peak and XPS verification of Cu(II) (935 eV) distinguish SCu NPs as hybrid coordination complexes rather than metallic Cu(0) particles, achieving a 1.92:1 SK: Cu molar ratio with 95.5% SK encapsulation efficiency-substantially surpassing the 20–50% loading in typical biosynthesized CuO NPs, where incomplete precursor utilization limits yields to ~ 40% in fungal-mediated syntheses^12^.This organic-inorganic synergy, evidenced by XRD amorphous SK matrix embedding nanoscale crystallites, mitigates cytotoxicity associated with inorganic CuNPs while enhancing bioavailability and environmental compatibility over AgNPs, positioning SCu NPs for scalable antimicrobial deployment^30^.

DFT simulations at the B3LYP/6-311 + G(d, p) level with SMD solvation elucidate SCu NPs’ structural robustness, predicting thermodynamically favored 1:1 (A1/A2, ΔG difference 0.85 kcal/mol) and 1:2 (B2, ΔG = -6.42 kcal/mol) Cu(II)-SK coordination modes via quinone/hydroxyl chelation, with IRI analysis revealing dominant ionic bonds augmented by van der Waals and hydrogen-bond networks that prevent reduction to Cu(0), diverging from polyphenol-driven syntheses^30^.The B2 configuration’s minimized HOMO-LUMO gap, indicative of heightened chemical softness per Koopmans’ theorem, implies amplified reactivity for ROS generation through ligand-to-metal charge transfer (LMCT, ~ 280–350 nm bands), paralleling Cu(II)-lawsone complexes where bidentate O, O-coordination yields stable octahedral geometries with water ligands, enhancing anti-inflammatory efficacy in vivo^28^.This computational-experimental alignment highlights electronic tunability in naphthoquinone-metal hybrids, where reduced bandgaps foster charge transfer for antimicrobial catalysis, as seen in juglone-copper chelates maintaining activity against Gram-positive strains via matrix interactions—yet underexplored in SK systems, warranting further EPR validation of coordination geometries^28^.

SCu NPs demonstrate superior antibacterial efficacy against S. aureus ATCC 25,923, with MIC/MBC values (4/8 µg/mL) halved relative to SK alone (8/16 µg/mL), outperforming reported SK MICs of 35–70 µg/mL against foodborne S. aureus and 7.8–125 µg/mL across MSSA/MRSA strains, attributable to synergistic Cu(II)-mediated ROS (Cu²⁺/Cu⁺ cycling at 0.158 V) amplifying SK’s membrane disruption and agr quorum-sensing inhibition^28^.Time-kill kinetics show 41% viability reduction in 2 h versus SK’s 57%, with SEM evidencing profound membrane collapse, exceeding standalone CuO NPs’ MICs (62.5–125 µg/mL biosynthesized, or 2.5–280 µg/mL ultra-small variants) by 15- to 600-fold against S. aureus, owing to nanoscale penetration (10–20 nm) breaching peptidoglycan barriers that impede ionic Cu in conventional formulations^45^.Antibiofilm assays quantify ~ 66% biomass reduction (OD570 ~ 0.17) in 2 h, surpassing 1,4-naphthoquinone metal chelates’ 50–60% inhibition at higher doses and copper-sorbate complexes’ reported antibiofilm potency (emphasizing structure-activity but without quantified percentages in S. aureus models), via multi-target ROS and adhesin disruption—mechanisms potentiated by Cu(II) coordination in quinone scaffolds^44^.This addresses biofilm-conferred 10- to 1000-fold antibiotic resistance, a hallmark of S. aureus infections^46^.

Collectively, SCu NPs embody a bioinspired countermeasure to AMR, projected to cause over 39 million cumulative deaths by 2050, offering cost-effective, stable alternatives to AgNPs with reduced cytotoxicity, as copper surfaces in clinical trials achieve 27–83% reductions in hospital-acquired infections^47^.Limitations encompass the moderate zeta potential, potentially requiring PEGylation for in vivo stability to avert off-target ROS effects on eukaryotes, and necessitate mechanistic probes into ROS specificity against MRSA biofilms in animal models^47^.Future trajectories should evaluate SCu NPs’ synergy with antibiotics (fractional inhibitory concentration indices < 0.5, mirroring SK-colistin pairings against MRSA) and integration into wound dressings, potentially transforming therapeutics for ICU-associated infections amid escalating AMR^48^.

## Conclusion

In this study, we successfully synthesized and characterized SCu NPs through a green chemistry approach, utilizing SK as both a chelating and stabilizing agent. The resulting SCu NPs, with a size range of 10–20 nm, demonstrated enhanced stability and superior antibacterial efficacy against *S. aureus* compared to SK alone. The integration of copper’s redox properties with SK’s bioactive framework not only amplified the antimicrobial potency but also introduced a novel hybrid nanomaterial with potential applications in combating bacterial infections and biofilms. Density functional theory (DFT) calculations provided mechanistic insights into the stable coordination of copper with SK, highlighting the structural basis for the observed reactivity. These findings underscore the promise of combining natural products with nanotechnology to develop advanced antimicrobial agents, offering a sustainable and effective strategy to address contemporary challenges in infectious diseases.

## Data Availability

The datasets used or analyzed during the current study are available from the corresponding author upon reasonable request.
